# Retraction: Polyphenolic fraction obtained from *Thalassia testudinum* marine plant and thalassiolin B exert cytotoxic effects in colorectal cancer cells and arrest tumor progression in a xenograft mouse model

**DOI:** 10.3389/fphar.2025.1639694

**Published:** 2025-06-12

**Authors:** Frontiers Editorial Office

The journal retracts the 30 November 2020 article cited above.

Following publication, concerns were raised regarding the integrity of the images in the published figures. After being contacted by the Editorial Office, the authors requested retraction of the cited article, stating that they were unable to provide raw data or a satisfactory explanation. The findings reported in the article are no longer supported by the data. An investigation was conducted in accordance with Frontiers’ policies that confirmed this; therefore, the article has been retracted.

This retraction was approved by the Chief Editors of Frontiers in Pharmacology and the Chief Executive Editor of Frontiers. The authors agree to this retraction.

Frontiers would like to thank Sholto David for contacting the journal regarding the published article.

